# Functional solid additive modified PEDOT:PSS as an anode buffer layer for enhanced photovoltaic performance and stability in polymer solar cells

**DOI:** 10.1038/srep45079

**Published:** 2017-03-24

**Authors:** Binrui Xu, Sai-Anand Gopalan, Anantha-Iyengar Gopalan, Nallal Muthuchamy, Kwang-Pill Lee, Jae-Sung Lee, Yu Jiang, Sang-Won Lee, Sae-Wan Kim, Ju-Seong Kim, Hyun-Min Jeong, Jin-Beon Kwon, Jin-Hyuk Bae, Shin-Won Kang

**Affiliations:** 1 School of Electronics Engineering, College of IT Engineering, Kyungpook National University, 80 Daehakro, Bukgu, Daegu, 41566, Korea; 2 Future Industries Institute, Division of Information Technology, Engineering and Environment, University of South Australia, Mawson Lakes, 5095, South Australia; 3 Research Institute of Advanced Energy Technology, Kyungpook National University, 80 Daehakro, Bukgu, Daegu, 41566, Korea; 4 Department of Chemistry Education, Kyungpook National University, 80 Daehakro, Bukgu, Daegu, 41566, Korea

## Abstract

Poly(3,4-ethylenedioxythiophene):poly(styrene sulfonate) (PEDOT:PSS) is most commonly used as an anode buffer layer in bulk-heterojunction (BHJ) polymer solar cells (PSCs). However, its hygroscopic and acidic nature contributes to the insufficient electrical conductivity, air stability and restricted photovoltaic (PV) performance for the fabricated PSCs. In this study, a new multifunctional additive, 2,3-dihydroxypyridine (DOH), has been used in the PEDOT: PSS buffer layer to obtain modified properties for PEDOT: PSS@DOH and achieve high PV performances. The electrical conductivity of PEDOT:PSS@DOH films was markedly improved compared with that of PEDOT:PSS. The PEDOT:PSS@DOH film exhibited excellent optical characteristics, appropriate work function alignment, and good surface properties in BHJ-PSCs. When a poly(3-hexylthiohpene):[6,6]-phenyl C_61_-butyric acid methyl ester blend system was applied as the photoactive layer, the power conversion efficiency of the resulting PSCs with PEDOT:PSS@DOH(1.0%) reached 3.49%, outperforming pristine PEDOT:PSS, exhibiting a power conversion enhancement of 20%. The device fabricated using PEDOT:PSS@DOH (1.0 wt%) also exhibited improved thermal and air stability. Our results also confirm that DOH, a basic pyridine derivative, facilitates adequate hydrogen bonding interactions with the sulfonic acid groups of PSS, induces the conformational transformation of PEDOT chains and contributes to the phase separation between PEDOT and PSS chains.

Bulk-heterojunction (BHJ) polymer solar cells (PSCs) have received extensive attention owing to their advantageous characteristics such as their light weight, flexibility, large area processability and low cost^1^. In the past few years, the power conversion efficiency (PCE) of BHJ PSCs has reached over ~11% through significant research progresses on the optimization of the materials, the inclusion of additives, and device engineering^2^. BHJ PSCs included with an interfacial layer or a buffer layer sandwiched between the electrode and the photoactive layer exhibited enhanced PV performances^3^. Literature reveals that buffer layer plays key roles in PSCs, such as providing Ohmic contacts with the photoactive layer (typically with a donor polymer), photoinduced carriers (electrons or holes) and work function (WF) tuning of the electrode^4^,^5^. Control of the electrical, optical, morphological, and conformational characteristics of the buffer layer is one of the most significant methods for enhancing the PV performances of PSCs. According to the charge-carrier extraction, the buffer layer is termed either as an anode buffer layer ((ABL) or hole transporting layer), which transports holes to the anode, or a cathode buffer layer, which transports electrons to the cathode^6^.

To date, polymeric buffer layer materials such as polyaniline-poly(styrenesulfonate)^7^, poly(3,4-ethylenedioxythiophene): poly(styrene sulfonate) (PEDOT:PSS)^8^, polythiophene^9^ and p-type metal oxides (e.g., MoO_3_, V_2_O_5_)^10^ have been particularly attractive as the ABL in PSCs. PEDOT:PSS is an important ABL in PSCs mainly owing to its virtue of solution based device fabrication process, high optical transmittance in the visible region, moderate thermal stability, good interfacial characteristics between the BHJ composite active layer and ITO anode and excellent hole transport capability. However, PEDOT:PSS has a few disadvantages. The commercially available PEDOT:PSS (Clevios P VP AI. 4083) stock contains large proportions of insulating PSS (PEDOT:PSS ratio of 1:6), which compromises the electrical conductivity of the PEDOT:PSS. Besides that, the acidic nature of PEDOT:PSS (pH between 1 and 2) corrodes the interface between the ITO and the PEDOT:PSS and causes the diffusion of Indium into the photoactive layer, to result in degradation of the device^11^. Thus, several approaches have been employed to modify the properties of PEDOT:PSS, including the incorporation of dopants/additives (e.g., ethylene glycol^12^, dimethyl sulfoxide^13^, ionic liquids^14^, or nanoparticles^15^), and the post-treatment of the PEDOT:PSS film with suitable materials (e.g., hexafluoroacetone^16^, surfactant^17^, salts^18^, and acids^19^).

In this study, we focus on the modification of the PEDOT:PSS properties by using a new multifunctional organic solid additive (2, 3-dihydroxypyridine, (denoted as DOH) or 2, 3-pyridinediol) for specifically improving the PSC performances and stability characteristics. The multifunctionality of DOH arises from its chemical structure and properties. DOH is a pyridine derivative (pKa 5, pH = 8.22) (Fig. 1(A)). The physical properties of DOH arise as the consequence of cyclic 6-π electron and presence of an electronegative nitrogen atom in the ring. The aromatic π electron system does not require the participation of the lone pair of a nitrogen atom and keeps the pyridine unit as basic. DOH behaves as a proton acceptor due to the presence of two hydroxyl groups. The vicinal diol groups in DOH can participate in intermolecular hydrogen bonding as well as form intermolecular hydrogen bond with other molecules. Bearing the multifunctional capabilities of DOH in mind, we envisaged our intention is to introduce DOH as an additive into PEDOT:PSS film and investigate the effect of modified PEDOT@DOH on the PV performance of BHJ PSC. As depicted in Fig. 1(A), we envisage the formation of intermolecular hydrogen bonding between the hydrogen atoms of the hydroxyl groups in the DOH molecule and the sulfonate or sulfonic acid groups of PSS. The hydrogen bonds generated between the PEDOT:PSS and DOH can decrease the Coulombic attractive forces between PEDOT and PSS chains and cause redistribution of PEDOT and PSS chains. As a consequence of these influences of DOH on PEDOT:PSS, we anticiapte modification in the conductivity of the PEDOT:PSS. Moreover, the probable interactions between the PEDOT:PSS and DOH can induce conformational transitions of the conducting PEDOT chains. For example, there can be a transition in the conformation of PEDOT chains from the compressed coil form to the expanded coil form (Fig. 1(B)). When such a conformational transition occurs in the DOH modified PEDOT:PSS (PEDOT:PSS@DOH), it can have an associated change from the less conductive benzenoid structure to the more conductive quinoid structure (Fig. 1(C)). As a result of the structural reorganization, charge carriers are expected to be transported more rapidly in the expanded coil or linear conformation^20^, improving the conductivity and hole carrier mobility. Also, the alkaline DOH can suppress the acidic nature of PEDOT:PSS and improve the device stability.

Herein, we present a proof-of-concept demonstration of the roles of DOH in a fabricated BHJ PSC through systematic experimental studies. We investigated the probable structural, morphological, and surface modifications in the PEDOT:PSS due to the inclusion of DOH. We fabricated the BHJ PSCs with the device configuration of glass/indium tin oxide (ITO)/PEDOT:PSS or PEDOT:PSS@DOH/poly(3-hexylthiohpene):[6,6]-phenyl C_61_-butyric acid methyl ester (P3HT:PC_61_BM)/zinc oxide nanocrystals (ZnO NCs)/Al (Fig. 1(D)) to authenticate the roles of DOH in improving the PCE of the BHJ PSC. To evaluate the device performance, the most actively studied photoactive blend system (P3HT:PC_61_BM) was utilized to fabricate the BHJ PSCs. Subsequently, we examined the influence of DOH on the important properties of the PEDOT:PSS film, such as the electrical conductivity, WF tuning at the ITO/PEDOT:PSS@DOH interface, surface morphology, topography, hole mobility across the ITO/PEDOT:PSS@DOH interface, optical transparency, PV performances and device stability. Our results clearly revealed the multiple roles of DOH and informed that the blending of mild basic DOH concurrently tunes the energy level alignment and surface texture, enhances the electrical conductivity and hole mobility, yields adequate optical transparency and improves PV performances. Typically, the PSCs fabricated with PEDOT:PSS@DOH (1.0 wt%) exhibited a maximum a PCE of 3.49%, which was ~20% improvement over the PCE (2.92%) of the device without DOH (pristine PEDOT:PSS). Furthermore, the device fabricated with DOH exhibited improved air and thermal stability. Thus, the inclusion of mild basic DOH in PEDOT:PSS film improved both the photovoltaic performances and the stability of the BHJ PSC.

## Results and Discussion

Studies have earlier been performed to suppress the acidic nature of PEDOT:PSS by employing a strong basess such as NaOH^21^ and KOH^22^. However, those strong bases adversely influenced the charge transport properties of PEDOT:PSS, reducing the efficiency and stability of the fabricated BHJ PSCs. In this work, DOH was chosen as a mild base with unique structural features to induce hydrogen bonding interactions with PEDOT:PSS as well as to facilitate the multiple roles of DOH such as influencing the conductivity, WF at the interface, surface properties, and hole mobility. Therefore, we systematically elucidate the (i) microstructural modifications in the PEDOT:PSS film by DOH, (ii) the influence of DOH on the properties of the PEDOT:PSS film, and (iii) the enhancements in the PSC performance characteristics due to the inclusion of PEDOT:PSS@DOH.

### Microstructural modifications in PEDOT:PSS by DOH

**Figure 1:**
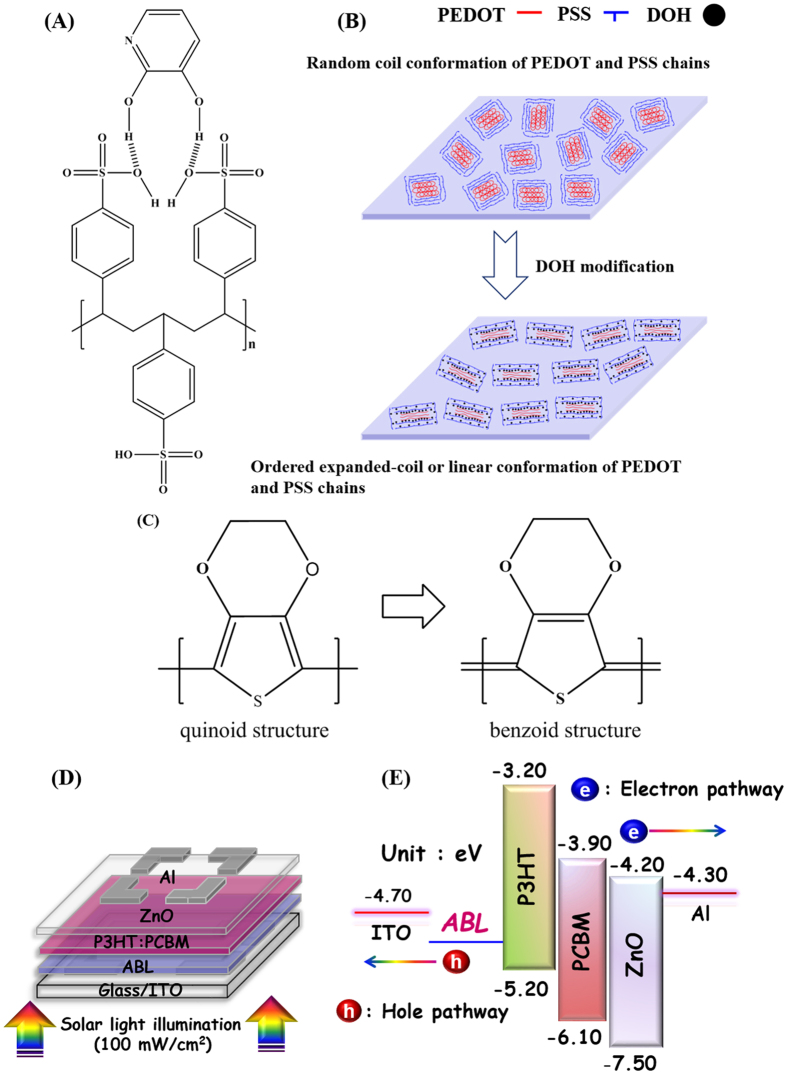
(**A**) Plausible mechanism of hydrogen bonding between DOH and PSS, (**B**) conformational transformation of PEDOT and PSS chains, (**C**) transformation of the PEDOT chain from the benzoid structure to the quinoid structure, (**D**) device schematic, (**E**) energy level band diagram.

**Figure 2:**
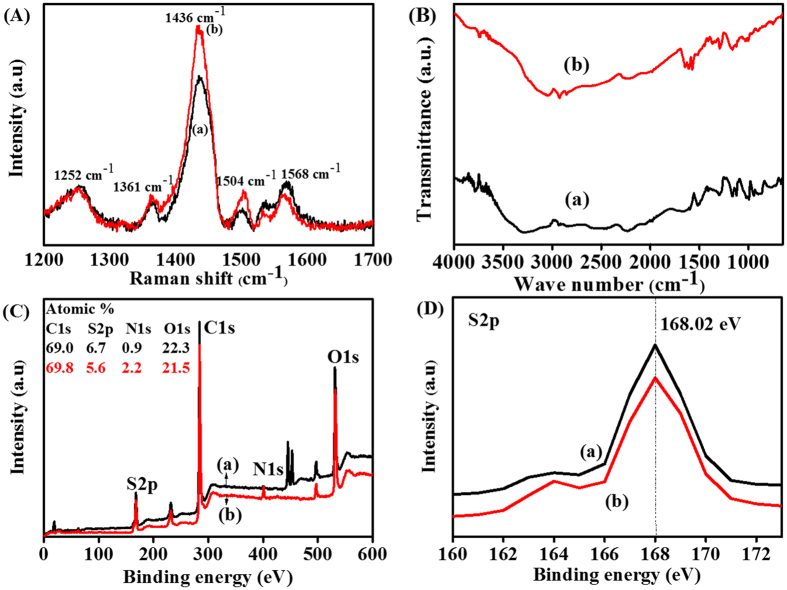
(**A**) Raman spectra of (a) pristine PEDOT:PSS and (b) PEDOT:PSS@DOH(1.0 wt%) films; (**B**) FTIR spectra of (a) PEDOT:PSS and (b) PEDOT:PSS@DOH(1.0 wt%) films. (**C**) XPS survey level O1*s*, C1*s*, N1*s*, and S2*p* spectra of (a) PEDOT:PSS and (b) PEDOT:PSS@DOH(1.0 wt%) films; (**D**) XPS core level S2*p* spectra of (a) PEDOT:PSS and (b) PEDOT:PSS@DOH(1.0 wt%) films.

Raman spectroscopy is a sensitive technique for investigating the conformational changes in polymers due to molecular-level interactions. The Raman spectra for pristine PEDOT:PSS and PEDOT:PSS@DOH(1.0 wt%) films are presented in Fig. 2(A). The most intense peak centered at 1,436 cm^−1^ is assigned to the C_α_ = C_β_ symmetric stretching vibration. The two other bands at 1,504 cm^−1^ (thiophene rings in the middle of PEDOT chains) and 1,568 cm^−1^ (thiophene rings at the end of PEDOT chains) correspond to the C_α_ = C_β_ asymmetric stretching vibrations^23^. The inconspicuous peak centered at 1,534 cm^−1^ arises from the splitting of the asymmetric vibrations. Two other peaks associated with the C_α_ − C_α_′ inter-ring stretching vibration and C_β_ − C_β_′ stretching vibration, respectively, were observed at 1,256 and 1,361 cm^−1^ 24. No additional peaks were observed in the Raman spectrum of the PEDOT:PSS@DOH(1.0 wt%) film (Fig. 2(A), curve b) compared with the Raman spectra of PEDOT:PSS (Fig. 2(A), curve a). However, the peak centered at 1,436 cm^−1^ was broadened and became more intense (Fig. 2(A), curve b) compared with the corresponding peak for the pristine film^25^ (Fig. 2(A), curve a). The relative intensity of the 1,436 cm^−1^ peak for PEDOT:PSS increased with the addition of DOH. These spectral changes in PEDOT:PSS@DOH(1.0 wt%) are correlated with the change in the conformation of the PEDOT chains from a benzoid structure to a quinoid structure. However, non-covalent hydrogen bonding can occur between the partially positive charged hydrogen atoms in the DOH and the partially negative charged oxygen in the SO_3_H groups of PSS (Fig. 1(A)). Thus, PEDOT chains can transform from a coiled conformation to an expanded-coil or a linear conformation^26^. Ouyang *et al*.^20^ proposed that the benzoid and quinoid structure of PEDOT chains (Fig. 1(B)) corresponds to the coiled conformation and expanded-coil or linear conformation, respectively (Fig. 1(B,C)). The expanded-coil conformation retains the two PEDOT rings in the same plane to facilitate the π-electron delocalization to cause increase in the conductivity of the PEDOT:PSS@DOH film^27^. Hence, we presume that the inclusion of DOH into PEDOT:PSS, is likely to provide additional charge carriers^28^. Additionally, the conformational transformation of PEDOT chains presumably can result in the enhanced hole mobility in the PEDOT:PSS@DOH film.

Fourier-transform infrared (FTIR) spectroscopy is a useful tool for investigating the structural changes due to covalent or non-covalent interactions between the two molecules through the functional groups present in them. Figure 2(B) presents the FTIR spectra for PEDOT:PSS and PEDOT:PSS@DOH(1.0 wt%) films. The C=C symmetric and asymmetric vibrations of the EDOT ring were centered at 1,426 and 1,561 cm^−1^, respectively. The band at 1,606 cm^−1^ is assigned to the C=C stretching vibration of the quinoid EDOT and phenyl side group. The band at 1,089 cm^−1^ is associated with the C–O–C stretching vibration of the EDOT ring. The bands at 834 and 903 cm^−1^ are attributed to the vibrational modes of the CS bond in the thiophene ring. The peak at 1,087 cm^−1^ is assigned to the stretching mode of the ethylenedioxy group. The bands at approximately 1,170 and 1,210 cm^−1^ represent the vibrational bands of the sulfonic groups –SO_2_ and –SO_3_ in the PSS^27^. Interestingly, a new peak was observed at 1,698 cm^−1^ for the PEDOT:PSS@DOH(1.0 wt%) (Fig. 2(B,C)x curve b) film, which indicates the possible hydrogen bonding interaction between DOH and PSS chains. The hydrogen bonding interactions can reduce the Coulombic attraction between PEDOT and PSS chains and cause phase separation between PEDOT and PSS as well as the reorganization of PEDOT chains into the expanded coil conformation (Fig. 2(B)). Furthermore, the interactions and inter-domain charge hopping between PEDOT chains can be reduced. The synergistic aspects of the expanded coil conformation and the interchain hopping can contribute to effective charge hopping to eventually result in improved conductivity^13^,^29^.

X-ray photoelectron spectroscopy (XPS) was performed to examine the changes in surface composition and chemistry involved in the PEDOT:PSS film due to the inclusion of DOH. Figure 2(C) reveal the existence of two types of S2*p* signals. The S2*p* doublet around 165–163 eV is assigned to the sulfur atoms in the PEDOT (Fig. 2(D))^30^. The higher binding energy peak at 168.05 eV corresponds to the sulfur atoms in PSS which arises due to withdrawal of the electron density at the sulfur atom by the three electronegative oxygen atoms of the –SO_3_H group. The integrated S2*p* peak area for PEDOT:PSS@DOH was lower as compared to PEDOT:PSS and suggested the possible phase separation between the PEDOT and PSS in PEDOT:PSS@DOH. This could lead to a decrease in PSS content on the film PEDOT:PSS@DOH surface. The lower content of elemental sulfur on the PEDOT:PSS@DOH(1.0 wt%) surface (5.6 atoms wt%) compared with that on the surface of pristine PEDOT:PSS (6.6 atomic wt%) (Fig. 2(C)) supports the phase separation and existence of lesser propotion of PSS on the suface of the PEDOT:PSS@DOH(1.0 wt%) film. The reduced PSS content on the surface increased the surface conductivity for the PEDOT:PSS@DOH.

**Figure 3:**
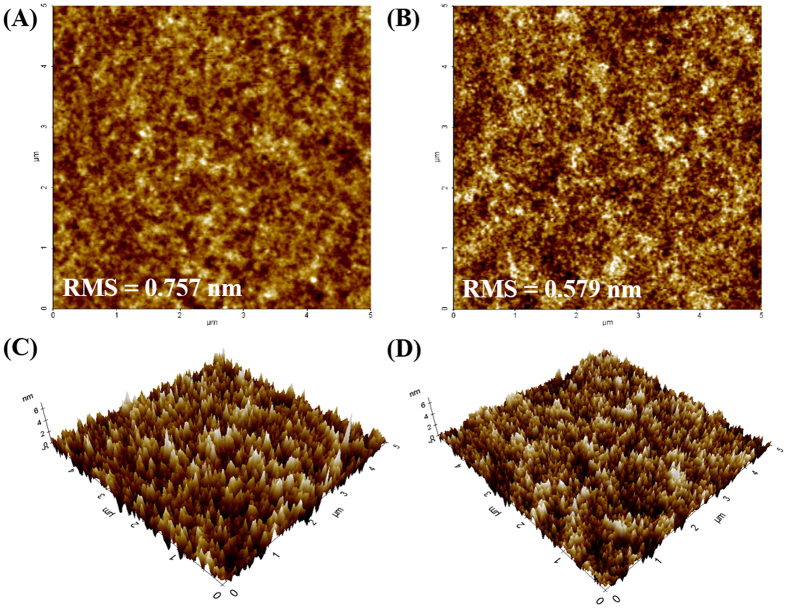
(**A**,**B**) 2D AFM images of PEDOT:PSS and PEDOT:PSS@DOH(1.0 wt%) films, (**C**,**D**) 3D AFM images of PEDOT:PSS and PEDOT:PSS@DOH(1.0 wt%) thin films.

To explore the effect of DOH on the topography of the PEDOT:PSS, atomic force microscopy (AFM) images of ITO/PEDOT:PSS and ITO/PEDOT:PSS@DOH(1.0 wt%) films were acquired (Fig. 3). In the phase images, the PEDOT:PSS grains formed by the PEDOT-rich core and PSS-rich shell appear as bright and dark regions, respectively^31^. An observable phase separation with full coverage on the substrate can be seen in the AFM image of the PEDOT:PSS@DOH(1.0 wt%) film, where the bright PEDOT-rich regions grew larger while the dark PSS-rich regions decreased in size, indicating that the surrounding insulating PSS-rich shell became smaller. For the pristine PEDOT:PSS and PEDOT:PSS@DOH(1.0 wt%) films, the root mean square roughness values were 0.757 and 0.579 nm, respectively^32^. These results indicate that the incorporation of DOH into the PEDOT:PSS rearranged the PEDOT and PSS chains, while the smoother buffer layer on top of the ITO improved the connection between the PEDOT-rich regions in both the parallel and vertical directions^33^. The phase separation between PEDOT and PSS chains provided efficient charge carrier hopping leading to superior hole transport pathways^34^.

### Influence of DOH on properties of PEDOT:PSS

#### Electrical conductivity

**Figure 4:**
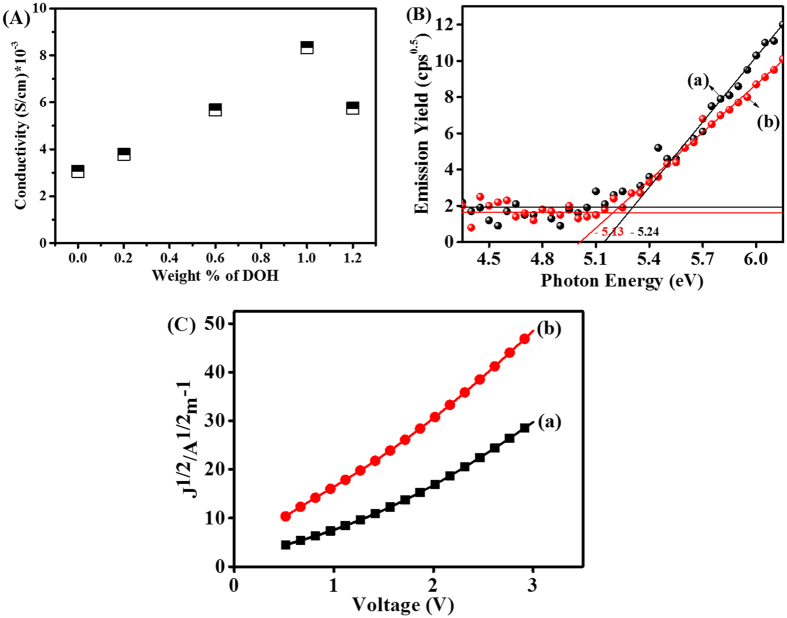
(**A**) Conductivity of PEDOT:PSS films with different concentrations of DOH, (**B**) PYS spectra of (a) PEDOT:PSS and (b) PEDOT:PSS@DOH(1.0 wt%), and (**C**) *J*^*1*/*2*^–*V* characteristics of the hole-only devices for (a) PEDOT:PSS and (b) PEDOT:PSS@DOH(1.0 wt%) based devices.

Figure 4(A) illustrates the variation in the electrical conductivity between the PEDOT:PSS and modified PEDOT:PSS (PEDOT:PSS@DOH) thin films based on nine measurements over different areas on the substrate. As shown in Fig. 4(A), the conductivity of the PEDOT:PSS@DOH (which is much higher than that of the pristine PEDOT:PSS film (3.06 × 10^−3^ S/cm)) increased as the DOH content increased from 0.2 to 1.0 wt% and reached the maximum of 8.33 × 10^−2^ S/cm for PEDOT:PSS@DOH(1.0 wt%). It was found that further incorporation of DOH beyond 1.0 wt% into the PEDOT:PSS did not increase the film conductivity^35^,^36^,^37^. The improved conductivity of PEDOT:PSS@DOH(1.0 wt%) is attributed to the conformational changes in the PEDOT chains (Fig. 1(B)). These were probably due to the redistribution of the PEDOT and PSS chains resulting from the hydrogen bonding between the PSS chains and DOH molecules (Fig. 1(B)), which reduced the proportion of the insulating PSS content in the PEDOT:PSS@DOH film^23^. The increase in the conductivity of the PEDOT:PSS@DOH(1.0 wt%) was expected to enhance the PCE of the PSCs.

### Electronic properties

The WFs of PEDOT:PSS and PEDOT:PSS@DOH films coated onto ITO substrates were evaluated using photoelectron yield spectroscopy (PYS). The WF was determined according to the point of intersection of the tangent line cutoff of the curve on the x-axis, where the y-coordinate was zero. The WFs of the PEDOT:PSS and PEDOT:PSS@DOH films were estimated as −5.24 and −5.13 eV, respectively, (Fig. 4(B)). Notably, the WFs of PEDOT:PSS and PEDOT:PSS@DOH(1.0 wt%) were substantially lower than the lowest unoccupied molecular orbital of PC_61_BM (−3.90 eV). Additionally, the WF of the PEDOT:PSS@DOH(1.0 wt%) (−5.13 eV) was far closer to the highest occupied molecular orbital (HOMO) energy level of P3HT (−5.10 eV) than to the HOMO level of pristine PEDOT:PSS (−5.24 eV). The closeness of the energy levels between the PEDOT:PSS@DOH(1.0 wt%) and P3HT is beneficial for better hole extraction from the HOMO energy level of P3HT to the ITO. The efficient hole extraction pathway minimizes the charge recombination and can contribute to the enhancement in the hole collection efficiency^4^,^5^.

### Hole mobility

Hole-only diodes were fabricated with a configuration of ITO/PEDOT:PSS or PEDOT:PSS@DOH(1.0 wt%)/P3HT:PC_61_BM/Au to determine the hole mobility. A high WF cathode (gold) was chosen to selectively extract hole carriers and to obtain hole-only mobility in a typical P3HT based blend system^38^. Additionally, the hole mobility of the hole-only device was relatively field and temperature-independent owing to the high degree of regioregularity and the purity of the polycrystalline material (P3HT)^39^,^40^. The hole mobility of the hole-only device was calculated at room temperature using the trap-filled space-charge-limited current model, according to the well-known Mott–Gurney equation^38^,^41^:


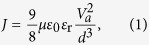



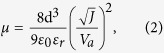


where *J* is the current density, *V*_*a*_ is the applied voltage, *d* is the active layer thickness (*d* = 214 nm determined by field-emission scanning electron microscopy (FE-SEM), Figure S1), *μ* is the charge carrier mobility, *ε*_*0*_ = 8.85 × 10^−12^ F/m is the dielectric permittivity with respect to free space, and *ε*_*r*_ = 3 is the relative dielectric constant of P3HT:PC_61_BM^42^. The slopes of the *J*^*1*/*2*^–*V* curves were calculated to be 10.13 and 15.34 for the PEDOT:PSS and PEDOT:PSS@DOH(1.0 wt%) based PSCs, respectively, as shown in Fig. 4(C). The hole mobility values at room temperature were estimated as 3.22 × 10^−4^ and 8.57 × 10^−4^ cm^2^/Vs, respectively^36^. The enhanced mobility was correlated with the improved conductivity of modified PEDOT:PSS arising from the conformational transformations of the PEDOT chains^43^.

### Photovoltaic performance

**Figure 5:**
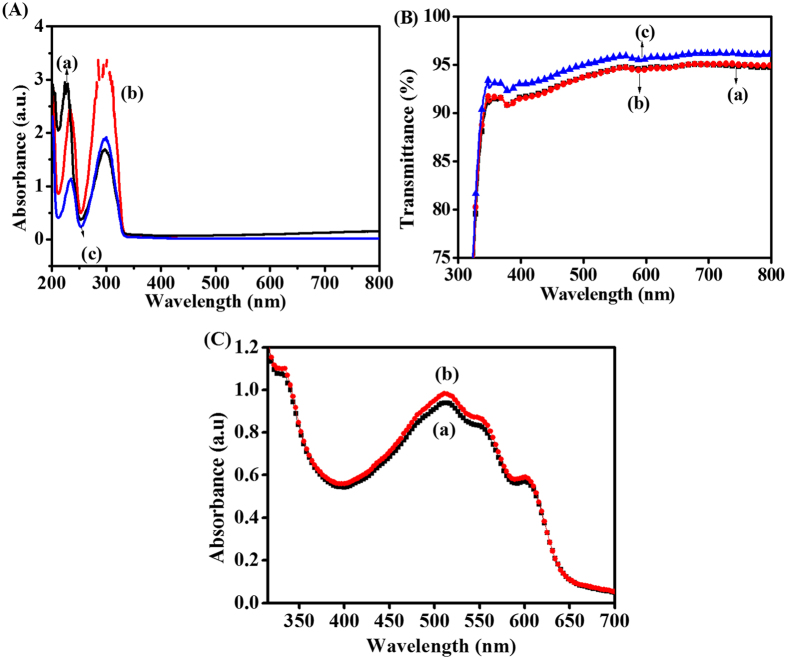
(**A**) UV–vis absorption spectra of (a) PEDOT:PSS, (b) PEDOT:PSS@DOH(1.0 wt%), and (c) DOH aqueous solution; (**B**) transmittance spectra of (a) PEDOT:PSS and (b) PEDOT:PSS@DOH(1.0 wt%) films coated on ITO substrates; (**C**) UV–vis absorption spectra of (a) PEDOT:PSS/P3HT:PC_61_BM and (b) PEDOT:PSS@DOH(1.0 wt%)/P3HT:PC_61_BM thin films.

After assessing the improvements in the electrical and electronic properties of PEDOT:PSS due to the inclusion of DOH, we intended to evaluate the PV performances of the device fabricated with PEDOT:PSS@DOH(1.0 wt%) as the ABL. The schematic of the PSC device configuration (Fig. 1(D)) and the energy level positions of the components (Fig. 1(E)) are presented. We investigated the optical transparency and properties of the PEDOT:PSS@DOH(1.0 wt%) in ITO/PEDOT:PSS@DOH(1.0 wt%) film in the PSC architecture. The UV-visible (UV-vis) spectra recorded for the aqueous solutions of PEDOT:PSS and PEDOT:PSS@DOH (1.0 wt%) are presented (Fig. 5(A)). The UV-vis spectrum of PEDOT:PSS exhibits peaks at 225 and 297 nm (Fig. 5(A), curve a). The spectrum for simple DOH displayed peaks at 235 and 298 nm (Fig. 5(A), curve b). Upon the inclusion of DOH in the PEDOT:PSS, (Fig. 5(A), curve c), the spectrum revealed the presence of two absorption peaks at 233 and 297 nm. The shifts in the peak (either a red shift (8 nm) from the peak of PEDOT:PSS or a blue shift (2 nm) from the peak of DOH) suggest probable interactions between the PEDOT:PSS and DOH.

The transmittance spectra of PEDOT:PSS and PEDOT:PSS@DOH(1.0 wt%) films coated on ITO were compared (Fig. 5(B)). The PEDOT:PSS@DOH(1.0 wt%) film exhibited significantly higher transmittances (>95%) in the visible region than that of the pristine PEDOT:PSS. The structural realignments or re-organization of the polymer chains, which are evident in the Raman spectra (Fig. 2(A)), FTIR (Fig. 2(B)) and the topographical changes revealed by AFM (Fig. 2(C)), are considered as the reasons for the increased transmittance for PEDOT:PSS@DOH(1.0 wt%) (Fig. 5(B))^41^. Figure 5(C) shows the UV-vis absorption spectra for ITO/PEDOT:PSS/P3HT:PC_61_BM and ITO/PEDOT:PSS@DOH(1.0 wt%)/P3HT:PC_61_BM. Four peaks are observed in the UV–vis spectra (Fig. 5(C)). The absorption peak at ~332 nm is assigned to PC_61_BM and three other absorption peaks at 511, 553, and 602 nm (two vibronic shoulders) correspond to P3HT^33^. These three peaks are attributed to the π–π* stacking transitions of P3HT^44^. Remarkably, the absorption spectrum of the ITO/PEDOT:PSS@DOH(1.0 wt%)/P3HT:PC_61_BM film indicates enhanced light absorption in the wavelength range from 320 to 610 nm as compared to the absorption of the ITO/PEDOT:PSS/P3HT:PC_61_BM film.

**Figure 6:**
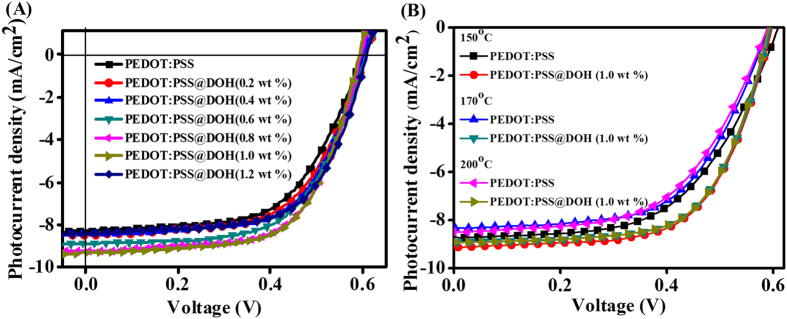
(**A**) Current density-voltage (*J*–*V*) characteristics measured for the BHJ-PSCs with different concentrations of DOH; (**B**) current density-voltage (*J*–*V*) characteristics measured for BHJ-PSCs with PEDOT:PSS and PEDOT:PSS@DOH(1.0 wt%) thermally annealed at 150, 170, and 200 °C.

To realize the efficacy of the PEDOT:PSS@DOH(1.0 wt%) film as an ABL in BHJ PSC, devices consisting of a P3HT:PC_61_BM blend system were fabricated. The schematic of the ITO/PEDOT:PSS or PEDOT:PSS@DOH(0.2, 0.4, 0.6, 0.8, 1.0, 1.2 wt%)/P3HT:PC_61_BM/ZnO NCs/Al device architecture and associated energy level diagram are presented in Fig. 1(D) and (E), respectively. The current density–voltage (*J*–*V*) curves of the fabricated PSCs with PEDOT:PSS@DOH(0.2, 0.4, 0.6, 0.8, 1.0, 1.2 wt%) are shown in Fig. 6(A). The PV performance parameters were recorded under ambient air conditions with illumination of 100 mW/cm^2^ air mass 1.5 (AM 1.5) and the PV performance parameters of the PSCs are summarized in Table 1. The fabricated PSCs with pristine PEDOT:PSS exhibited a PCE of 2.92% with open-circuit voltage (V_oc_), J_sc_, FF, and R_s_ values of 0.599 V, 8.272 mA/cm^2^, 0.58, and 183.01 Ω, respectively. However, an enhanced PCE (3.49%) was observed for the device with PEDOT:PSS@DOH(1.0 wt%) as the ABL, with J_sc_ of 9.309 mA/cm^2^, V_oc_ of 0.593 V, FF of 0.63, and R_s_ of 121.27 Ω. The nearly 20% improvement in the PCE (from 2.92% to 3.49%) was mainly due to the increase in J_SC_ and decrease in R_S_ caused by the addition of 1.0 wt% DOH. Further addition of DOH beyond 1.0 wt% in the PEDOT:PSS film decreased the PV performances as compared to devices with a lesser DOH content (upto 1.0 wt%). Nevertheless, the PCE (3.26%) of the PEDOT:PSS@DOH(1.2 wt%) based PSC was superior to that of the pristine PEDOT:PSS (2.92%). To clarify the reproducibility of the data, the performance statistics based on eight fabricated PEDOT:PSS and PEDOT:PSS@DOH based PSCs from two different batches are shown in Table 1. When PEDOT:PSS@DOH(1.0 wt%) was used as the ABL in the BHJ PSCs, the enhanced hole mobility was beneficial for efficient hole extraction/collection from the active layer to the ABL, improving the J_sc_ and the PV performance. The increase in the electrical conductivity for the PEDOT:PSS@DOH(1.0 wt%) film is ascribed to the decrease in the proportion of insulating PSS (5.6 atomic %) on the top surface for the PEDOT:PSS@DOH(1.0 wt%), which enabled the formation of continuous PEDOT domains, leading to an increase in J_sc_ (from 8.272 mA/cm^2^ to 9.309 mA/cm^2^) for the fabricated PSCs with the PEDOT:PSS@DOH(1.0 wt%) film as the ABL^45^.

### Thermal stability

In addition to the PV performance, the thermal stability is a critical parameter for the practical application of devices. Therefore, the thermal stability/durability of the PEDOT:PSS and PEDOT:PSS@DOH based PSCs were examined by subjecting the devices to heat treatment at 150 °C, 170 °C, and 200 °C for 10 min after the film deposition. For the PEDOT:PSS based device, when the annealing temperature of the device increased from 150 °C to 200 °C, concomitant reductions in J_sc_ and FF were observed, along with a drastic increase in R_s_ (Fig. 6(B) and Table 2). In contrast, for the PEDOT:PSS@DOH(1.0 wt%) device, a stable increasing trend was observed for the PV performance parameters: V_oc,_ J_sc,_ and FF. The R_s_ was minimized at 200 °C for the PEDOT:PSS@DOH(1.0 wt%) based device. The overall PCE of the PEDOT:PSS devices decreased by 0.09% at 170 °C and 0.17% at 200 °C. In contrast, the PCE of the PEDOT:PSS@DOH(1.0 wt%) based device showed only a marginal decrease, e.g., 0.04% at 170 °C. Importantly, the PEDOT:PSS@DOH(1.0 wt%) based device exhibited nearly stable device performance beyond 170 °C. A clear increase in the FF of the device with PEDOT:PSS@DOH(1.0 wt%) was observed when the annealing temperature increased. The excellent thermal stability of the PEDOT:PSS@DOH(1.0 wt%) device is attributed to the better phase separation between the PEDOT and PSS chains after the DOH modification^46^. The better thermal stability of the PEDOT:PSS@DOH(1.0 wt%) based PSCs suggests that the incorporation of DOH is beneficial for thermoelectric applications^47^,^48^,^49^.

### Evaluation of air stability

In our previous work, we demonstrated that pristine PEDOT:PSS based devices exhibited a diminished air stability due to the corrosion/degradation of the electrode^4^,^5^. In comparison, the PSCs fabricated with PEDOT:PSS@DOH(1.0 wt%) as the ABL exhibited a significantly improved air stability under ambient condition at room temperature (Figure S2). This is attributed to the neutralization effect and oxygen insensitivity of the basic DOH (a derivative of pyridine (pk_a_ = 5.22)).

## Conclusion

We demonstrated an efficient and new approach for improving the performance and stability of the PSCs by using an ABL included with a multifunctional organic additive (2, 3 dihydroxypyridine, DOH) in the PEDOT:PSS film. The PEDOT:PSS@DOH film exhibited superior properties, such as an enhanced conductivity, a WF shift from −5.24 to −5.13 eV, an increased hole mobility, and adequate transmittance, which synergistically improved the PV performances of the resulting device. The Raman and FTIR spectra revealed the transformation of PEDOT chains into a more conductive quinoid form with an expanded coil conformation. AFM results suggested that the PEDOT chains were separated from the PSS chains, that supported improving the conductivity and superior hole transport pathways in the PEDOT:PSS@DOH film. Furthermore, because of the reorganization of the PEDOT and PSS chains, ITO/PEDOT:PSS@DOH and ITO/PEDOT:PSS@DOH/P3HT:PC_61_BM films exhibited superior optical properties. The BHJ PSC with PEDOT:PSS@DOH (1.0 wt%) film as a buffer layer exhibited an excellent 20% increase in the PCE compared with a PEDOT:PSS based device. Moreover, the physicochemical properties and alkaline nature of DOH contributed to the significant enhancements of the thermal and air stability of the fabricated PSCs. Our results demonstrate the potential of PEDOT:PSS modification with mild basic DOH towards improving the PCE and the device stability. Thus, the present study paves the way for fabricating efficient and thermally stable BHJ PSCs.

## Experimental Section

### Materials

PEDOT:PSS (Clevios P VP AI. 4083) was purchased from Baytron, H.C. Starck, Inc. P3HT (molecular weight (M_W_) = 4,500) and PC_61_BM (M_W_ = 910.88) were obtained from Luminescence Technology Corp., Taiwan. 1,2-dichlorobenzene (DCB) (anhydrous, 99%) was purchased from Sigma–Aldrich and DOH was purchased from Tokyo Chemical Industry Co., Ltd. and used as received.

### Preparation of PEDOT:PSS@DOH

Different weight ratios of DOH (0.2, 0.4, 0.6, 0.8, 1.0 and 1.2 wt%) were separately blended into the PEDOT:PSS aqueous solution. The mixture was stirred overnight at 40 °C to form a homogenous solution and then utilized as the ABL in the BHJ PSCs.

### Device fabrication

Glass/ITO/PEDOT:PSS or PEDOT:PSS@DOH/P3HT:PC_61_BM/ZnO NCs/Al devices were fabricated (Fig. 1(B)). ITO glass substrates (3 × 3 cm^2^) with a sheet resistance of 10 Ω cm^−2^ were pre-cleaned using acetone, deionized water, acetone, and isopropanol in an ultrasonic cleaner for 10 min, sequentially. The PEDOT:PSS and PEDOT:PSS@DOH solutions were filtered through a 0.45-μm filter before being deposited onto pre-cleaned ITO substrates at 3000 rpm for 30 s and subsequently annealed on a hot plate at 150 °C for 10 min. To form a photoactive layer, P3HT (20 mg) and PC_61_BM (20 mg) (1:1 wt%) were dissolved in DCB with stirring for 12 h at 60 °C in the dark to maximize the mixing of the blend components. Then, the blend system was spin-coated onto ITO/ABL with a thickness of ~214 nm (Figure S1). Next, the photoactive blend films were dried in a plastic petri dish for 1 h. A thin protective layer film (ZnO NCs) was formed by drop-casting and the resulting film was thermally annealed under vacuum conditions at 100 °C for 5 min. Next, a ~150 nm aluminium electrode was thermally evaporated at a pressure of 10^−6^ Torr. The active area of the fabricated PSCs was 9 mm^2^ (defined by the shadow mask of the vertical overlap between the ITO and aluminum electrodes). All the film deposition steps were performed in open air with a humidity of 15–25%.

### Device characterization

The PV performance of the PSCs was measured in ambient air using a solar simulator (XES-300S1, SAN-EI Electric Corp., Japan) with a 300-W Xenon arc lamp under standard AM 1.5G illumination (100 mW cm^−2^) and a computer-controlled source meter (2400 series, Keithley, Inc.). The air stability of the PSCs was investigated according to standards of the International Summit on Organic and Hybrid Photovoltaic Stability (ISOS-D-1 shelf) under ambient conditions^50^.

### Physico-chemical characterization

A four-point contact geometry (Model CMT-SR1060N, Changmin Tech Co Ltd) in the Van der Pauw configuration was carried out at room temperature to determine the film conductivity of the PEDOT:PSS and PEDOT:PSS@DOH thin films. Gold probes were placed in direct contact with the substrate. The reported electrical conductivity (average) values were obtained from nine measurements performed in different regions of the substrate. The WFs of the PEDOT:PSS and PEDOT:PSS@DOH(1.0 wt%) films spin-coated on ITO glass were determined using PYS (Model AC-2, Riken-Keiki, Japan) with a deuterium UV lamp via the low-energy-electron counter method in the energy range of 3.4–6.2 eV. Solution-state UV-vis absorption, optical transmittance, and UV-vis absorption spectra were obtained using a UV-vis spectrophotometer (Shimadzu Corp., Japan). AFM images (two-dimensional (2D), thre-dimensional (3D) revealing the polymer film morphology were acquired using a scanning probe microscope (Model 5500, Agilent Technologies, Inc.) and analyzed with the software Park Systems XEI. An inVia confocal Raman microscope (Renishaw) based on charge coupled device detection was used to obtain the Raman scattering spectra for PEDOT films with a 632-nm excitation laser line. The FTIR spectra were collected using an FTIR/NIR spectrophotometer (Model Frontier, PerkinElmer, USA) with a deuterated triglycine sulfate detector after 64 scans. XPS was performed using a VG Microtech MT-500 ESCA system with Al Kα X-ray radiation (1486.6 eV). The thickness of the PEDOT films were measured using FE-SEM (Hitachi SU8220, Japan).

## Additional Information

**How to cite this article**: Xu, B. *et al*. Functional solid additive modified PEDOT:PSS as an anode buffer layer for enhanced photovoltaic performance and stability in polymer solar cells. *Sci. Rep.* **7**, 45079; doi: 10.1038/srep45079 (2017).

**Publisher's note:** Springer Nature remains neutral with regard to jurisdictional claims in published maps and institutional affiliations.

## Supplementary Material

Supplementary Information

## Figures and Tables

**Table 1:** Summary of photovoltaic performance parameters for PEDOT:PSS and different loadings of DOH into the PEDOT:PSS buffer layer.

Loading of DOH into PEDOT:PSS (wt%)	V_oc_ (V)	J_sc_ (mA/cm^2^)	FF	R_s_ (Ohm)	PCE^a^ (%)	PCE^b^ (%)
0	0.60	8.272	0.58	183	2.92	2.74
0.2	0.60	8.320	0.60	172	2.99	2.78
0.4	0.60	8.428	0.61	152	3.17	2.94
0.6	0.61	8.866	0.61	151	3.27	3.03
0.8	0.60	9.224	0.63	123	3.47	3.21
1.0	0.60	9.309	0.63	121	3.49	3.25
1.2	0.60	8.794	0.63	130	3.26	3.01

The average PCE values were obtained from eight independent devices. PCE^a^ and PCE^b^ are the best and average PCE values obtained from eight devices.

**Table 2:** Summary of photovoltaic performance parameters with different thermal annealing temperatures for PEDOT:PSS and PEDOT:PSS@DOH(1.0 wt%) buffer layer.

Anode buffer layer	Annealing temperature (°C)	V_oc_ (V)	J_sc_ (mA/cm^2^)	FF	R_s_ (Ohm)	PCE^a^ (%)	PCE^b^ (%)
PEDOT:PSS	150	0.61	8.504	0.59	167	2.99	2.77
PEDOT:PSS@DOH		0.60	9.127	0.63	121	3.44	3.19
PEDOT:PSS	170	0.60	8.340	0.58	177	2.90	2.69
PEDOT:PSS@DOH		0.60	8.925	0.64	123	3.40	3.14
PEDOT:PSS	200	0.60	8.311	0.56	197	2.82	2.61
PEDOT:PSS@DOH		0.59	8.920	0.65	117	3.40	3.17

The average PCE values were obtained from eight independent devices. PCE^a^ and PCE^b^ are the best and average PCE values obtained from eight devices.
