# Corrigendum: Functional solid additive modified PEDOT:PSS as an anode buffer layer for enhanced photovoltaic performance and stability in polymer solar cells

**DOI:** 10.1038/srep46779

**Published:** 2017-05-04

**Authors:** Binrui Xu, Sai-Anand Gopalan, Anantha-Iyengar Gopalan, Nallal Muthuchamy, Kwang-Pill Lee, Jae-Sung Lee, Yu Jiang, Sang-Won Lee, Sae-Wan Kim, Ju-Seong Kim, Hyun-Min Jeong, Jin-Beom Kwon, Jin-Hyuk Bae, Shin-Won Kang

Scientific Reports 7: Article number: 4507910.1038/srep45079; published online: 03 24 2017; updated: 05 04 2017

The original version of this Article contained a typographical error in the spelling of the author Jin-Beom Kwon, which was incorrectly given as Jin-Beon Kwon. This has now been corrected in both the PDF and HTML versions of the Article.

