# Supplementary material for: Functional solid additive modified PEDOT:PSS as an anode buffer layer for enhanced photovoltaic performance and stability in polymer solar cells
Source: Sci Rep. 2017 Mar 24;7:45079. doi: 10.1038/srep45079 (PMC5364469; doi:10.1038/srep45079)
Supplement: Supplementary Information [file srep45079-s1.doc]

**Supplementary Information**

**Functional solid additive modified PEDOT:PSS as an anode buffer layer for enhanced photovoltaic performance and stability in polymer solar cells**

Binrui Xu^1#^, Sai-Anand Gopalan^1,2#^, Anantha-Iyengar Gopalan^3,4^, Nallal Muthuchamy^4^, Kwang-Pill Lee^3,4^, Jae-Sung Lee^1^, Yu Jiang^1^, Sang-Won Lee^1^, Sae-Wan Kim^1^, Ju-Seong Kim^1^, Hyun-Min Jeong^1^, Jin-Beon Kwon^1^, Jin-Hyuk Bae^1^, and Shin-Won Kang^1*^

^1^School of Electronics Engineering, College of IT Engineering, Kyungpook National University, 80 Daehakro, Bukgu, Daegu 41566, Korea

^2^Future Industries Institute, Division of Information Technology, Engineering and Environment, University of South Australia, Mawson Lakes 5095, South Australia

^3^Research Institute of Advanced Energy Technology, Kyungpook National University,

80 Daehakro, Bukgu, Daegu 41566, Korea

^4^Department of Chemistry Education, Kyungpook National University,

80 Daehakro, Bukgu, Daegu 41566, Korea

Correspondence and requests for materials should be addressed to S.W.K*

(email: [swkang@knu.ac.kr](mailto:swkang@knu.ac.kr)) or G.S (email: [SaiAnand.Gopalan@unisa.edu.au](mailto:SaiAnand.Gopalan@unisa.edu.au))

^#^Authors contributed equally to this work


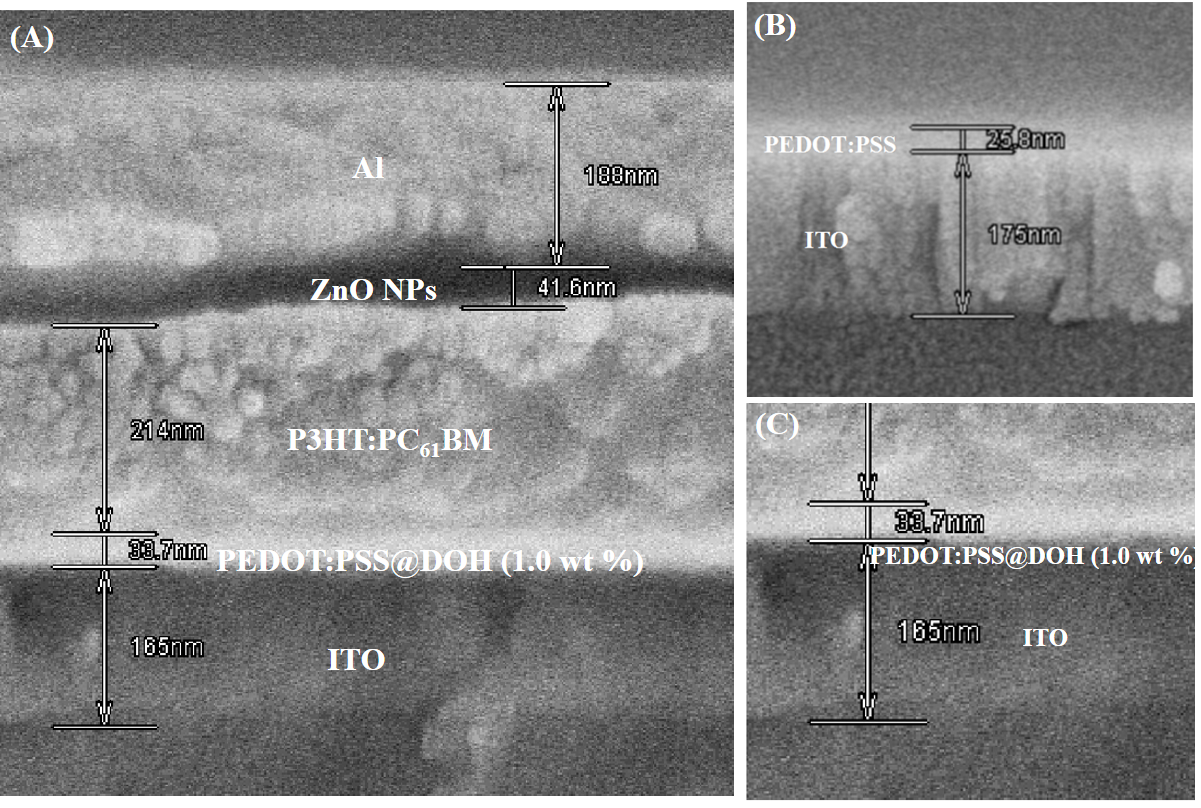


**Figure S1.** FE-SEM images of (A) the completed device with structure of glass/ITO/PEDOT:PSS@DOH (1.0 wt %)/P3HT:PC_61_BM/ZnO NCs/Al), (B) ITO/PEDOT:PSS and (C) ITO/PEDOT:PSS@DOH (1.0 wt %).

**Figure S2.** Normalized air-stability characteristics of BHJ-PSCs with (A) PEDOT:PSS and (B) PEDOT:PSS@DOH (1.0 wt %) over 200 h of continuous testing.

The air-stability is one of the significant parameters associated with practical utilization. To further conform the properties of the PEDOT:PSS@DOH in BHJ-PSCs, the air-stability of devices with ITO/PEDOT:PSS or PEDOT:PSS@DOH(1.0 wt %)/P3HT:PC_61_BM/ZnO NPs/Al was investigated under ambient conditions and as shown in Figure S2. The power conversion efficiency (PCE) and other performance parameters of the BHJ-PSCs were normalized, as shown in Figure S2. After 204 h, the PCEs of device with the pristine PEDOT:PSS and the device with PEDOT:PSS@DOH (1.0 wt %) were decayed 90 % and 78 % from their initial PCE, respectively. Furthermore, after 204 hours, the J_sc_ of the pristine device reduced by 63 % and the FF dropped by 71 %. In contrast, ~38 % of the J_sc_ and ~65 % of FF were dropped in the device with PEDOT:PSS@DOH(1.0 wt %). Simultaneously, the V_oc_ of both devices showed a similar linear trend. The decreased PCE in both BHJ-PSCs is related to the obviously reduced J_sc_ and FF. In conclusion, the device with PEDOT:PSS@DOH(1.0 wt %) exhibited better air-stability and a longer lifetime than the pristine device. The improved air-stability is related to the better phase separation between the PEDOT and PSS chains after DOH modification, and the PSS aggregations protected the PEDOT domains from oxygen . In addition, the pH value of the pristine PEDOT:PSS solution (1.7) and the PEDOT:PSS@DOH(1.0 wt %) solution (2.3) was measured using a Mettler Toledo benchtop pH meter. Another reason for the improved air stability of the completed BHJ-PSCs is the increasing pH value induced by the alkalinity of DOH [^3^](#_ENREF_3). By increasing the pH value, the damage to the device caused by PSS was controlled.

**References**

1 Savva, A. *et al.* Photovoltaic analysis of the effects of PEDOT:PSS-additives hole selective contacts on the efficiency and lifetime performance of inverted organic solar cells. *Solar Energy Materials and Solar Cells* **132**, 507-514 (2015).

2 Peters, C. H. *et al.* High Efficiency Polymer Solar Cells with Long Operating Lifetimes. *Advanced Energy Materials* **1**, 491-494 (2011).

3 Wu, S. *et al.* pH-neutral PEDOT:PSS as hole injection layer in polymer light emitting diodes. *Organic Electronics* **12**, 504-508 (2011).
